# Flavonoids: Overview of Biosynthesis, Biological Activity, and Current Extraction Techniques

**DOI:** 10.3390/plants12142732

**Published:** 2023-07-23

**Authors:** Sergio Liga, Cristina Paul, Francisc Péter

**Affiliations:** 1 Biocatalysis Group, Department of Applied Chemistry and Engineering of Organic and Natural Compounds, Faculty of Industrial Chemistry and Environmental Engineering, Politehnica University Timisoara, Carol Telbisz 6, 300001 Timisoara, Romania; sergio.liga96@gmail.com (S.L.); francisc.peter@upt.ro (F.P.); 2 Research Institute for Renewable Energies, Politehnica University Timisoara, Gavril Muzicescu 138, 300501 Timisoara, Romania

**Keywords:** natural compound, bioactive compounds, flavonoids, biological activity, current extraction techniques

## Abstract

Recently, increased attention has been paid to natural sources as raw materials for the development of new added-value products. Flavonoids are a large family of polyphenols which include several classes based on their basic structure: flavanones, flavones, isoflavones, flavonols, flavanols, and anthocyanins. They have a multitude of biological properties, such as anti-inflammatory, antioxidant, antiviral, antimicrobial, anticancer, cardioprotective, and neuroprotective effects. Current trends of research and development on flavonoids relate to identification, extraction, isolation, physico-chemical characterization, and their applications to health benefits. This review presents an up-to-date survey of the most recent developments in the natural flavonoid classes, the biological activity of representative flavonoids, current extraction techniques, and perspectives.

## 1. Introduction

In recent years, important research efforts have focused on the exploitation and use of natural compounds in the production of new products as well as the development of processes on an industrial scale.

In this respect, flavonoids, a class of natural polyphenolic compounds, have attracted continuously increasing attention. In addition to their widespread nature, they also exhibit a multitude of biological activities, making them exciting for many scientific fields. Flavonoids are secondary metabolites of plants that contain a benzo-γ-pyrone skeleton in their structure, being produced by various synthesis pathways, namely the phenylpropanoid pathway, the shikimate pathway, and the flavonoid pathway [1,2,3,4]. 

Their physico-chemical parameters, biological activity, and bioavailability are closely related and conferred based on the chemical structure. Based on the structure of flavonoids and depending on the change in the main structure, flavonoids can be classified into six major categories: (i) flavanones, (ii) flavones, (iii) isoflavones, (iv) flavonols, (v) flavanols, and (vi) anthocyanins [5,6]. Vegetables and fruits are an indispensable natural resource of flavonoids, but current research has shown that microorganisms, such as fungi and bacteria, can also produce flavonoids from plant biomass [7]. 

**Figure 1:**
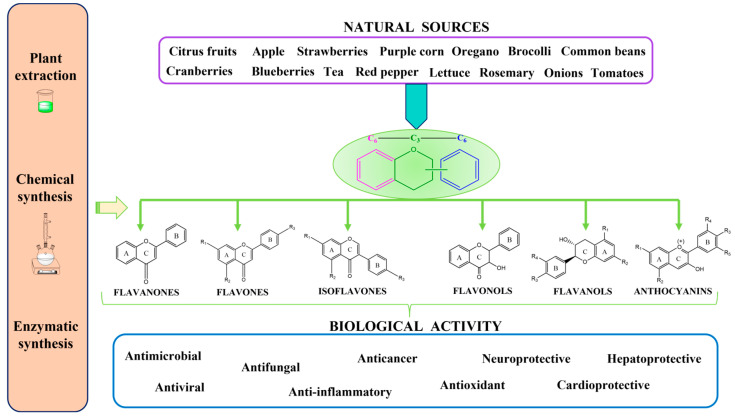
Natural sources of flavonoids, obtaining possibilities, and biological activity [1,2,3,4].

Recent research has directed on the extraction, isolation, and characterization of these compounds from different plant families, leading to progression from traditional extraction methods (e.g., maceration, decoction, percolation, and Soxhlet extraction) to the development of modern, environmentally friendly extraction techniques (e.g., microwave-assisted extraction, ultrasound-assisted extraction, supercritical fluid extraction, pressurized liquid extraction, matrix solid-phase dispersion, pulsed electric field extraction, and enzyme-assisted extraction), which could also have industrial applicability in several sectors such as the food, pharmaceutical, and cosmetic industries [1,8,9]. The main natural sources of flavonoids, the possibilities of obtaining them, and the biological activities are summarized in Figure 1. 

## 2. Biosynthesis of Flavonoids

Flavonoids are secondary metabolites of plants obtained from primary metabolic precursors and are generated via various biosynthetic pathways. 

The shikimate pathway involves several key enzymes and six-step reactions for the biosynthesis of shikimic acid, starting with the aldol condensation reaction of phosphoenolpyruvic acid and D-erythrose 4-phosphate [1,4]. Chorismic acid, the end product of the shikimate pathway, is converted into the amino acid phenylalanine through the action of prephenate-aminotransferase (PhAT) and arogenate-dehydratase (ADT) enzymes [4].

The phenylpropanoid pathway also plays a major role in the biosynthesis of flavonoids, starting from the amino acid phenylalanine [1,2,3,4]. In the presence of phenylalanine-ammonia liase (PhAAL), phenylalanine is desaminated to form trans-cinnamic acid [3,4]. Next, under the action of cinnamate-4-hydroxylase (C4L), it converts trans-cinnamic acid into 4-coumaric acid. It will provide, under the action of 4-coumarate-CoA-ligase (C4CoAL), the compound 4-coumaroyl-CoA, which plays a crucial role in the biosynthesis of flavonoids via the phenylpropanoid pathway, the production of the coumarin skeleton, and the initiation of the flavonoid pathway [1,2,3,4]. The condensation reaction between 4-coumaroyl-CoA with three molecules of 3-malonyl-CoA, under the action of the enzyme chalcone-synthase, yields 2′,4′,6′,4-tetrahydroxy chalcone [1,2,3,4]. Under the action of chalcone-flavanone isomerase, this compound is further isomerized in flavanone, initiating the flavonoid pathway that will produce the different classes of flavonoids. 

**Figure 2:**
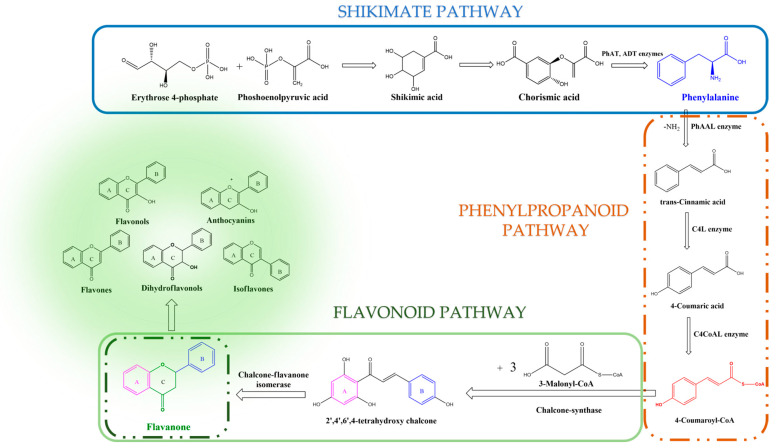
Overview of the main steps of the flavonoid biosynthesis pathway [1,2,3,4].

The shikimate, phenylpropanoid, and flavonoids pathways are summarized in Figure 2.

Flavonoids are derivatives of 2-phenyl-benzo-γ-pyrone (2-phenyl-3,4-dihydro-2H-1-benzopyran-4-one), being included in the large family of natural polyphenolic compounds with structure type C3-C6-C3 [1]. This basic structure contains, as seen in Figure 2, two aromatic benzene rings (A and B) connected by the heterocyclic pyrane ring (C) that contains an oxygen atom. Chalcones, which do not contain that third ring (C), are generally considered precursors of the different flavonoid classes [2]. Depending on the degree of nucleus oxidation, the saturation level of the segment C_3_, and the place of substituents insertion, there are several classes of flavonoid compounds, mainly classified as flavanones, dihydroflavonols, flavones, isoflavones, flavonols, and anthocyanins [1,2,3,4]. Flavonoids are found in plants in free form (aglycones) or linked to sugars (glycosylated flavonoids) [1,2,3,4,5,6,10].

## 3. Classification and Biological Activity 

### 3.1. Flavanones

Flavanones or 2-arylchroman-4-ones are obtained by the flavonoid pathway, with isomerization of 2′,4′,6′,4-tetrahydroxychalcone, in the presence of chalcone-flavanone isomerase [1,5,6,10]. Flavanones can be found in different plant families, such as *Compositae*, *Fabaceae,* and *Rutaceae*. 

Depending on the type of plant, flavanones can be isolated from vegetative parts (rhizomes, stem, leaves, flowers, and fruits) and generative organs (branches, bark, and roots) [10]. Citrus fruits (especially grapefruit), peppermint, licorice, tomatoes, and associated aliments (fruit juices and peeled fruits) are a major dietary source of flavanones [6,10]. 

**Figure 3:**
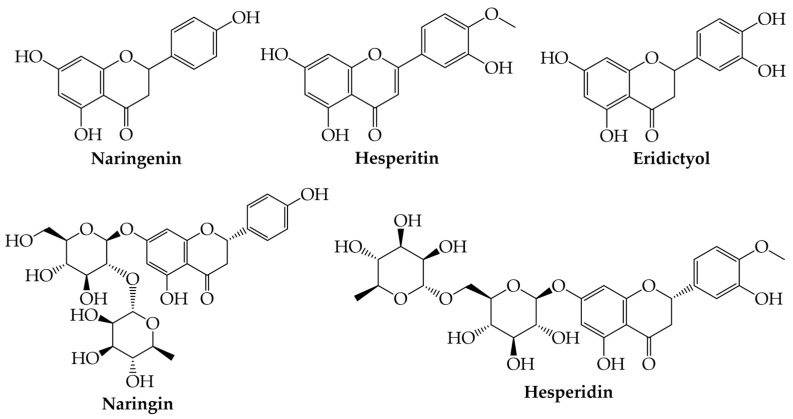
Examples of flavanones.

Flavanones can be found as forms of aglycones or as complexes with O- or C-glycosides [1,2,3,4,5,6,10]. Some examples of such compounds are naringenin (aglycon), naringin (glycoside), hesperitin (aglycon), hesperidin (glycoside), and eriodictyol (Figure 3) [5,10]. 

Naringenin, or 5,7-dihydroxy-2-(4-hydroxyphenyl)-chroman-4-one, is a citrus flavanone that has various biological activities and has been studied as a potential biological compound for the treatment of a variety of diseases. For example, Wei et al. [11,12] found that naringenin treatment reduced atrial fibrosis in different species of rats with induced cardiovascular disease. Additionally, Chtourou et al. [11,13] reported that naringenin in hepatocytes decreases pro-inflammatory cytokines. According to the literature, naringenin treatment decreased cancer cell proliferation and induced cell apoptosis in breast cancer [14,15,16], prostate cancer [14,15,17], lung cancer [14,15,18], and colon cancer [14,15,19]. Furthermore, Tutunchi et al. [20] have shown that naringenin may be considered a promising treatment strategy against COVID-19.

Hesperidin, or hesperetin-7-O-rutinoside, is used for different biological activities, such as the treatment of type 2 diabetes; antioxidant, anti-inflammatory, anticancer, and antiviral effects; biofilm protection; and protection against cardiovascular disorders [21,22,23,24,25].

Eriodictyol, or 2-(3,4-dihydroxyphenyl)-5,7-dihydroxy-2,3-dihydrochromen-4-one, and its glycoside Eriocitrin were reported in the literature to have a broad spectrum of biological activities, such as protection against cardiovascular issues; skin protection; antitumor, antioxidant, and anti-inflammatory activity; and immunomodulatory and hepatoprotective effects [26,27,28].

### 3.2. Flavones

Flavones, or 2-aryl-4H-chromen-4ones, are synthesized by the dehydrogenation of flavanones, generating a double bond between positions C-2 and C-3, under the action of a group of enzymes known as flavone-synthases [1,2,3,4]. 

Depending on their prevalence in nature, most flavones can be found in all parts of plants, in the form of compounds obtained by methylation, glycosylation, hydroxylation, acylation, or other modifications [29]. They are the primary pigments in white flowers and realize the co-pigmentation effect with anthocyanins in blue flowers [29,30]. 

**Figure 4:**
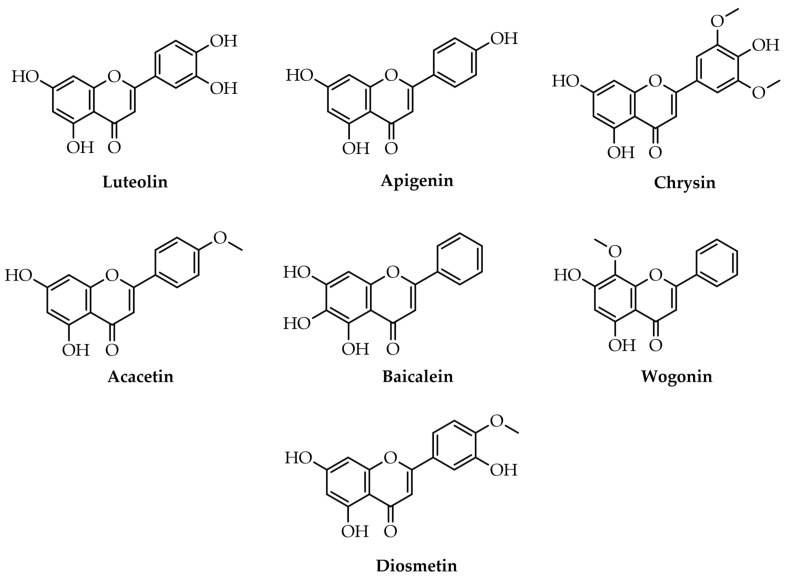
Examples of flavones.

The most widespread flavones are apigenin, luteolin, chrysin, acacetin, baicalein, wogonin, and diosmetin (Figure 4) [1,29,30,31]. Luteolin and apigenin are widespread in grains, vegetables, and medicinal herbs, being considered the most representative in food sources [29,30].

Several biological activities of flavones have been reported in the scientific literature, such as abiotic and biotic protection and anti-inflammatory, antimicrobial, and anticancer activities [29,31]. For example, Liu R. et al. have shown that in mouse models with induced amnesia, apigenin ameliorates spatial learning and memory deficits, protects microvessel integrity, and attenuates neuronal loss [32]. A systemic review has shown the biological potentials of baicalein and wogonin against ischemia-induced neurotoxicity and damage in the brain and retina [31,33].

### 3.3. Isoflavones 

**Figure 5:**
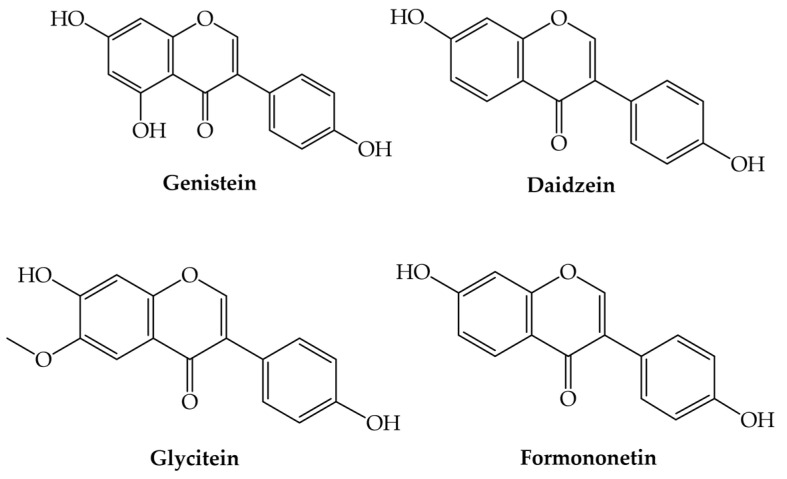
Examples of isoflavones.

Isoflavones, or 3-aryl-4H-chromen-4ones, are synthesized from flavanones under the action of two enzymes: isoflavone-synthase and hydroxy-isoflavanone dehydratase [1,2,3,4,34,35,36]. According to the literature, isoflavones are included in the large group of nutraceuticals [36]. The most common isoflavones are genistein, daidzein, glycitein, and formononetin (Figure 5).

Soybeans and other leguminous plants are the main sources of isoflavones [37]. According to their molecular structure, isoflavones represent one of the most common categories of phytoestrogens, are similar in particular to 17-β-estradiol, manifest different biological activities—especially fungistatic, antibacterial, antiviral, and antioxidant—prevent angiogenesis, and exert estrogenic and/or antiestrogenic effects [34,38,39]. 

### 3.4. Flavonols

**Figure 6:**
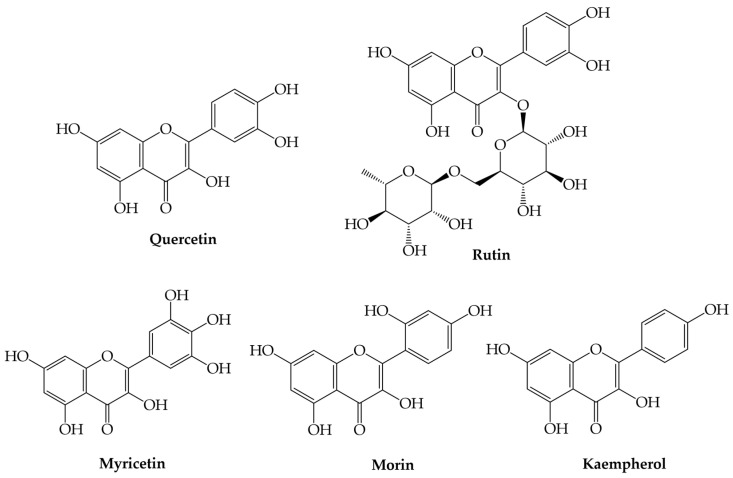
Examples of flavonols.

Flavonols are hydroxylated at position C-3 of ring C by flavonol synthase [1,2,3,4]. Quercetin, rutin, myricetin, kaempferol, and morin are some popular flavonols found in a wide variety of foods (Figure 6).

A systemic chapter showed the anti-inflammatory efficacy of flavonols against rheumatoid arthritis [40]. Quercetin, or 3,3,4,5,7-pentahydroxyflavone, is abundantly found in nature, being one of the most widely occurring polyphenols. Quercetin protects the body against oxidative stress by downregulating the level of malondialdehyde and scavenging several free radicals (e.g., hydrogen peroxide, superoxide, and hydroxyl radicals) [41,42]. Chen et al. [41,43] showed that quercetin increased cell IFN-γ expression and decreased interleukine-4 positive cell expression. Some other studies demonstrated that quercetin exerts an effect on cancer cells by inducing extrinsic and intrinsic pathways of apoptosis and autophagy [41,44]. Another research study revealed that quercetin’s antimicrobial activity disrupts cell membrane integrity, inhibits nucleic acid synthesis, and inhibits biofilm formation [45]. 

Rutin (3,3′,4′,5,7-pentahydroxyflavone-3-rhamnoside), or vitamin P, is a flavanol glycoside derivative of quercetin found in various medicinal plants and food sources. In a study carried out by Sui et al., rutin was shown to exhibit anti-inflammatory activity by negatively regulating Rho-related coiled-coil protein kinase signaling by promoting the expression of cystathionine-β-synthase and effectively inhibiting the inflammatory progress of osteoarthritis [46]. Li et al. reported that rutin inhibits ox-LDL-mediated macrophage inflammation and foam cell generation, which are both associated with autophagy activation [47]. In addition, rutin is known to have anti-atherosclerotic, antiallergic, anti-inflammatory, and antiviral properties [48,49].

### 3.5. Flavanols

**Figure 7:**
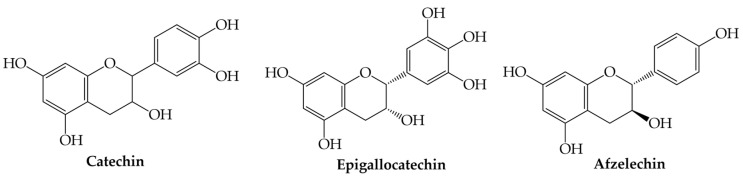
Examples of flavanols.

Flavanols are a subgroup of flavonoids characterized by the absence of a double bond between the carbon atoms C_2_ and C_3_ in the (C) ring, while featuring a hydroxyl group (s) in C_3_ or C_4_. Four types of flavanols have been found in nature: (i) flavan-3-ols, (ii) flavan-4-ols, (iii) isoflavan-4-ols, and (iv) flavan-3,4-diols [1,2,3,4,50]. The most common flavanols are catechin, epigallocatechin, and afzelechin (Figure 7).

The biological properties of flavanols have been extensively studied to reveal their anti-inflammatory, anticancer, antiviral, antimicrobial, and cardioprotective properties [50,51,52]. The most common sources of flavanols are cocoa and green tea, and there are numerous studies that have shown that health-promoting effects have been attributed to these natural compounds [53].

### 3.6. Anthocyanins

Anthocyanins are the glycosylated forms of the corresponding aglycones named anthocyanidins and are formed of a flavylium cation backbone hydroxylated in different positions under the action of dihydroflavonol-reductase and leucoanthocyanidin dioxygenase [1,2,3,4,54,55]. They are natural pigments responsible for the color of plants (blue, red, and purple) and can be found in all plant tissues, including leaves, flowers, and fruits. The color and stability of anthocyanins are influenced by pH, metal ions, light, and temperature [54,56]. Anthocyanins became of interest as natural therapeutic compounds because they have the ability to suppress neuroinflammation and support antioxidant activity, antimicrobial activity, antitumor activity, and immune function [55,56].

**Figure 8:**
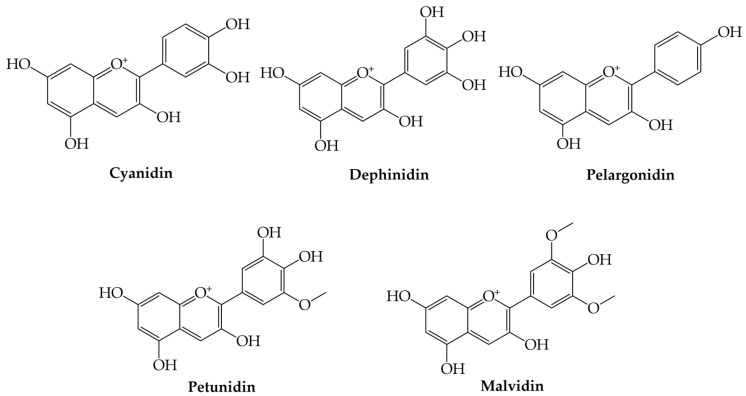
Examples of the most common anthocyanins.

The most common anthocyanins are cyanidin, delphinidin, pelargonidin, petunidin, and malvidin (Figure 8).

Samarpita et al. showed that cyanidin can be used as a small-molecule drug to treat patients with rheumatoid arthritis because it suppressed IL-17A, a cytokine found in the serum and synovial fluid of patients with rheumatoid arthritis [57]. Another study by Wang et al. showed that cyanidin ameliorated CCl4-induced liver injury in mice by improving the activity of antioxidant enzymes such as SOD and CAT and by decreasing the level of oxidative products such as TNF-α, IL-β, and IL-6. They also pointed out that with administration of cyanidin, the protein levels of NF-κB, a regulator of inflammation, and its downstream genes were significantly reduced [58]. 

Wu et al. identified the molecular mechanism in which delphinidin inhibited the viability of HER-2-positive breast cancer cell lines by decreasing the protein expression level of p-c-Raf, p-MEK1/2, and p-ERK1/2 and regulating the protein expression level of Bax and Bcl-2 and also inhibited the activation of NF-κB and nuclear translocation of NF-κB/p65 by inducing phase arrest and apoptosis of G_2_/M [59]. Kang et al. revealed that combined treatment by delphinidin with γ-ionizing radiation enhanced apoptotic cell death, activated the JNK/MAPK pathway, and effectively improved antiproliferative effects by increasing radiation sensitivity in A549 cells (human non-small cell lung cancer) by upregulation of autophagy after radiation therapy [60]. 

Cremonini et al. investigated the effects of supplementation with a cyanidin- and delphinidin-rich extract on postprandial dysmetabolism, inflammation, and redox and insulin signaling, triggered by the consumption of a high-fat meal. The study revealed that the extract rich in cyanidin and delphinidin reduced postprandial increases in other markers of inflammation, such as lipopolysaccharides binding protein plasma concentration and TNFα levels in peripheral blood mononuclear cells, as well as those of cardiometabolic outcomes (plasma levels of glucose, triglycerides, and cholesterol) [61]. 

In Table 1, we summarize some of the most important biological activities of the main representatives of the six classes of flavonoids.

## 4. Actual Limitations of Extended Utilization of Flavonoids

The actual limitations of the use of flavonoids in medical, pharmaceutical, food, and cosmetic fields are governed by two major issues: (i) chemical and biophysical properties, such as low solubility, chemical stability, bioavailability, and pharmacokinetics through metabolic stability (hepatic, intestinal, and intestinal microflora); (ii) plant production, such as very low yield of these secondary metabolites of plants relative to biomass and difficulties regarding the improvement of biosynthesis and complicate isolation, extraction, and purification methods [98]. 

**Figure 9:**
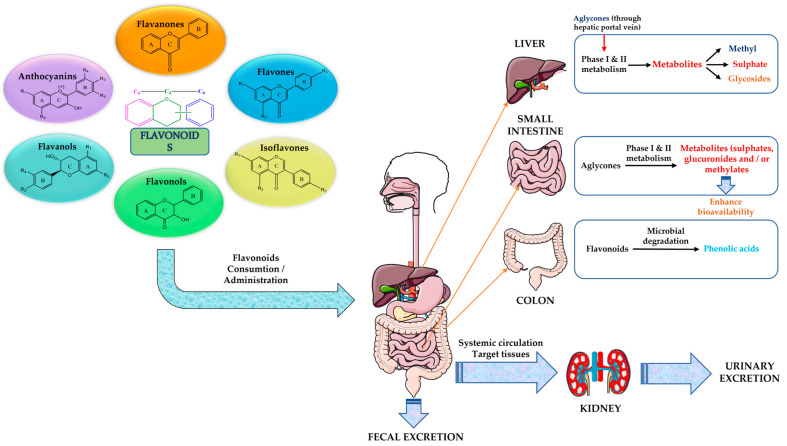
Limitations in the bioavailability of flavonoids in the human body.

One of the main limitations of flavonoids is represented by their pharmacokinetic properties within the human body (Figure 9). 

One of the main concerns regarding flavonoids is their low bioavailability. After oral administration, a small percentage of flavonoids are absorbed in the upper gastrointestinal tract (oral cavity) and a significant amount can reach the small intestine and can also interact with the intestinal microbiota (colon). Others may be metabolized under the action of liver metabolizing enzymes (cytochrome *P450*) to generate active metabolites [98,99,100,101]. 

The absorption rate of flavonoids in the human body is different due to the conformation of the molecular structure and pH values. After being absorbed by the intestinal epithelium, flavonoids are transformed into conjugated metabolites, namely glucuronides, sulphates, and methylated metabolites, first in the intestine and then in the liver.

The aglycones of flavonoids, with a small molecular structure and high hydrophobicity, enter through the hepatic portal vein in the liver, where two main metabolic reactions occur: the oxidation reaction by cytochrome P450 enzymes (Phase I) and the binding reaction (Phase II) [99,100]. Flavonoid glycosides possess higher hydrophilicity and molecular weight and can only be absorbed after being hydrolyzed to aglycone or phenolic acid by the intestinal microbiota. There are four main types of cleft rings in the colon: (i) flavones and flavanones to form C6-C3 phenolic acids; (ii) flavonols to form C6-C2 phenolic acids; (iii) flavanols to form C6-C3 phenols; (iv) isoflavones to form derivatives of ethylphenols [99,100]. Flavonoid–microbiome interactions may also prove helpful in the treatment of different diseases [100,101,102,103,104]. Rapid metabolic elimination of flavonoids highlights the need to develop novel pathways to improve their delivery.

## 5. Current Extraction Techniques

**Figure 10:**
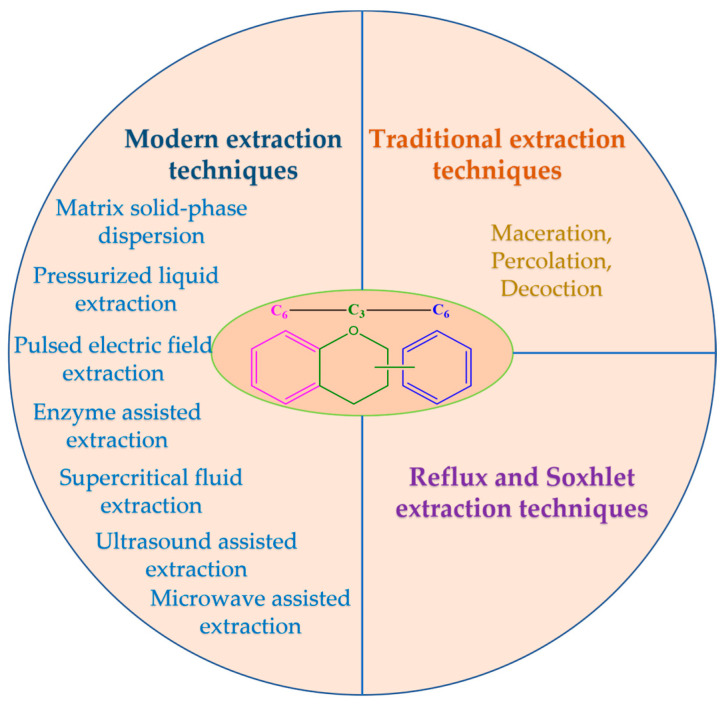
Main extraction techniques of flavonoids [8,9].

Selection of the most appropriate method for the extraction of flavonoids from different plants is often difficult and depends on several factors, such as the stability of the flavonoids, the nature of the solvents, the amount of extract required, and the appropriate techniques and equipment used for extraction. However, the most important extraction options can be classified into three groups: conventional or traditional techniques, reflux and Soxhlet techniques, and recently developed (modern) techniques (Figure 10).

### 5.1. Traditional Extraction Techniques

Traditional extraction techniques are most often used over time because they do not require special equipment and large amounts of product can be obtained. 

Maceration is the most common extraction technique, with the disadvantages of a longer extraction time and lower extraction selectivity and efficiency compared to other methods [8,9]. Maceration is applied to the extraction of vegetable products containing active ingredients that are slightly soluble in a temperature-proper solvent. As a working method, plant products are treated with a specified volume of solvent (e.g., methanol, ethanol, acetone, or water). They are kept in contact with the solvent for a fixed and variable time, mainly between 12 h and a few days. After this step, the process is followed by filtration. Other disadvantages of the maceration process include the use of large amounts of solvent and the need to purify the extract.

Percolation is more efficient than maceration as an exhaustive extraction technique, being a continuous process in which the solvent and the plant material flow in opposite directions and the solvent is constantly replaced by fresh solvent [105]. 

The decoction extraction technique may not be used to extract thermolabile or volatile components due to the high processing temperatures and has the disadvantage of many water-soluble impurities present in the extract [105]. The decoction process is used on plant products with stronger plant walls, such as rhizomes, roots, and bark.

### 5.2. Reflux and Soxhlet Extraction Techniques

The Soxhlet extraction technique represents a combination of percolation and maceration methods, where the plant product is positioned in a porous cotton thimble-holder, being gradually filled with condensed fresh solvent from a distillation flask during the whole process. Once the level of fresh solvent reaches above the siphon bend, the solvent flows into the flask through the siphon tube and is repeatedly unloaded back into the distillation flask, carrying the extracted analytes in the bulk liquid until extraction is considered complete [106]. The Soxhlet technique involves smaller amounts of solvent and a shorter extraction time compared to traditional extraction techniques, being used only for extracts that contain thermostable flavonoids.

Reflux extraction is a technique more commonly used compared to percolation and decoction and represents an extraction process at a constant temperature with repeatable evaporation and condensation of the solvent [107].

Some reports on such extraction techniques will be reviewed in more detail. Babich et al. used Soxhlet extraction with methanol to obtain biologically active substances (luteolin-7-glucoside, acacetin, apigenin-7-O-glucoside, and hesperetin) from *G. glabra*. The methanol extracts of *G. glabra* obtained by the Soxhlet method exhibited the highest antibacterial activity against *E. coli*, *P. aeruginosa*, and *B. subtilis* [108]. In a study carried out by Nuzul et al., high amounts of total phenolics (107.65 ± 0.01 mg GAE/g) and flavonoids (43.89 ± 0.05 mg QE/g) were obtained from Bambusa beecheyana using the Soxhlet method and methanol as the solvent. Moreover, the extract exhibited strong antioxidant activity compared to ascorbic acid, with an IC_50_ value of 40.43 μg/mL [109]. Yuan Ma et al. [110] used reflux extraction to obtain polyphenols from the shell of *Pleioblastus amarus* (Keng) and showed that the best extraction parameters were an ethanol concentration of 75%, a 20:1 liquid to solid ratio, and an extraction time of 2.1 h. Sati et al. showed that reflux extraction was the most efficient technique for the recovery of flavonoid (quercetin, kaempferol, and isorhamnetin) glycosides from Ginkgo biloba, as well as for obtaining the highest antimicrobial and antioxidant activities [86]. 

### 5.3. Modern Extraction Techniques

The extraction methods described above have several disadvantages, such as the use of large amounts of solvent, long processing times, occasional loss of solvents by evaporation, low selectivity, and the necessity of purifying the extract. To overcome these bottlenecks, advanced extraction techniques have been developed with many advantages, including prevention of pollution, avoidance of the concentration phases of the extract, reduction of solvent consumption, and the possibility of automation [8,9].

#### 5.3.1. Microwave-Assisted Extraction

**Figure 11:**
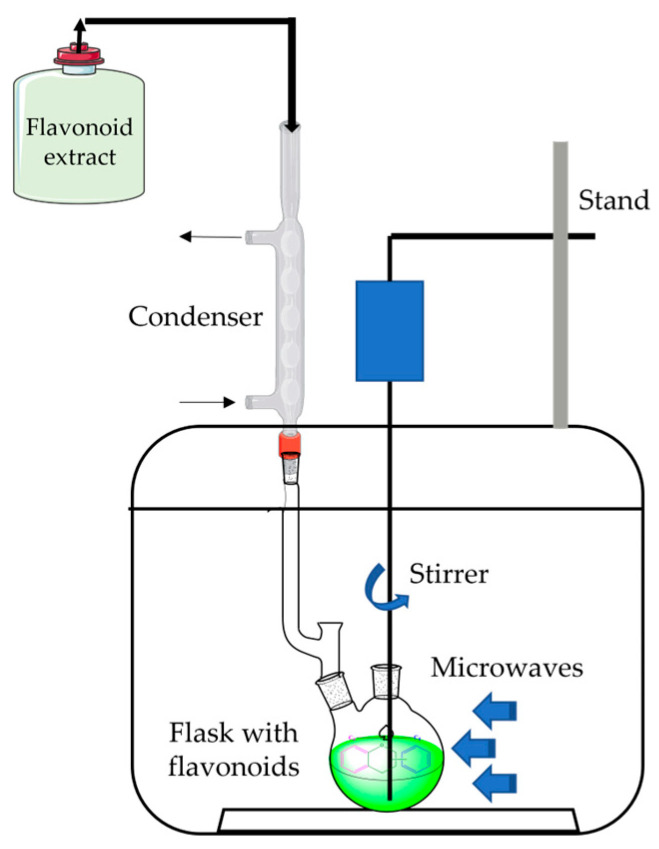
Schematic model of the microwave-assisted extraction apparatus.

Microwave-assisted extraction is a selective technique that uses microwave energy to heat solvents in contact with a sample to partition analytes from the sample matrix into the solvent [8,9,111]. It has advantages such as shorter time and a higher extraction rate, fewer solvent requirements, and lower costs (Figure 11). 

In a study conducted by Niu et al., microwave-assisted extraction was used to extract flavonoids from the leaves of *Alpinia oxyphylla* Miq. The optimal extraction conditions were determined as follows: 50% ethanol concentration, 1:20 solid–liquid ratio, 70 °C temperature, cycle index of 3. Under the optimized conditions, the extraction yield of total flavonoids was 28.24% [112]. Zhao et al. showed the potential of microwave-assisted extraction to extract epicatechin gallate (GAE) from the fruit of *Melastoma sanguineum*. The optimal extraction conditions were 31.33% ethanol and 45 min extraction time at 52.24 °C and 500 W, resulting in the highest value of total phenolic content of 39.02 ± 0.73 mg GAE/g dry weight. Furthermore, microwave-assisted extraction significantly reduced the amount of organic solvent and the extraction time compared to Soxhlet extraction [113]. Choommongkol et al. used microwave-assisted extraction to recover 2′,4′-dihydroxy-6′-methoxy-3′,5′-dimethyl-chalcone, a flavonoid with anticancer activity, from *Syzygium nervosum* fruit. Compared to other solvents, ethanol produced the highest flavonoid yield at 1298 ± 5 μg/g dry weight [114]. 

#### 5.3.2. Ultrasound-Assisted Extraction

**Figure 12:**
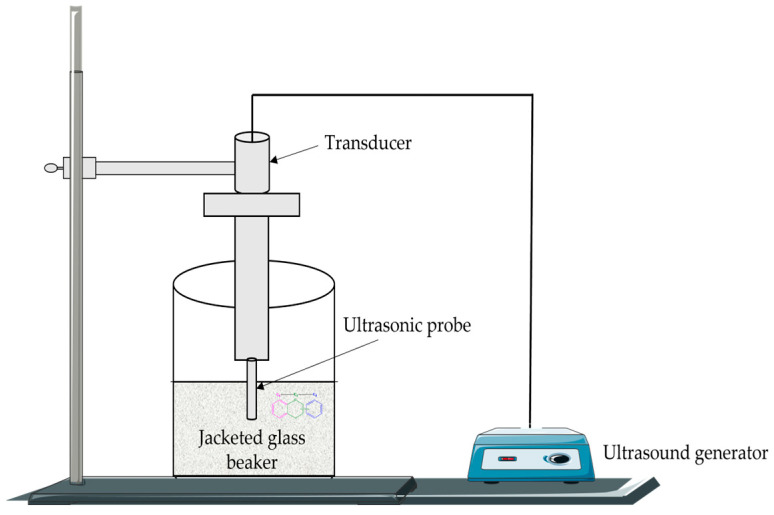
Schematic model of the ultrasound-assisted extraction apparatus.

The ultrasound-assisted extraction technique, or sonication, consists of the use of ultrasound energy in the form of waves and solvents to extract target bioactive compounds from various plant matrices [8,9,115]. Ultrasound waves generate small vacuum bubbles in the liquid, resulting in high temperatures and pressures [115]. Ultrasonic power is an important parameter that affects the extraction yield of flavonoids. Although increasing the ultrasonic power is obviously beneficial for the extraction yield, it should also be noted that excessive ultrasonic energy can produce damaging effects on flavonoids (Figure 12).

Several recent studies reported excellent results using this technique. Pham et al. developed an efficient ultrasound-assisted procedure for the extraction of flavonoids from *C. hindsii* leaves. A maximum total flavonoids content of 23.6 mg QE/g was obtained using 130 W ultrasonic power, 40 °C extraction temperature, 29 min extraction time, and 65% ethanol concentration [116]. Mai et al. also optimized the ultrasound-assisted extraction conditions but using the response surface methodology method to extract antioxidants from ‘Jinfeng’ kiwifruit. Optimal conditions were established as 68% ethanol concentration, 20 mL/g liquid/solid ratio, 30 min extraction time, 42 °C extraction temperature, and 420 W ultrasonic power. Under these optimal conditions, the ABTS value of the kiwifruit extract was 18.5% higher compared to that obtained by conventional solvent extraction [117]. Gueffai et al. demonstrated that basically the same parameters, extraction time, temperature, and solvent concentration had a significant impact on the phenolic compounds content in black cumin defatted extracts. The total phenolic content of the product obtained by ultrasound-assisted extraction under optimal conditions was significantly higher than that extracted by the conventional technique [118]. 

#### 5.3.3. Supercritical Fluid Extraction

**Figure 13:**
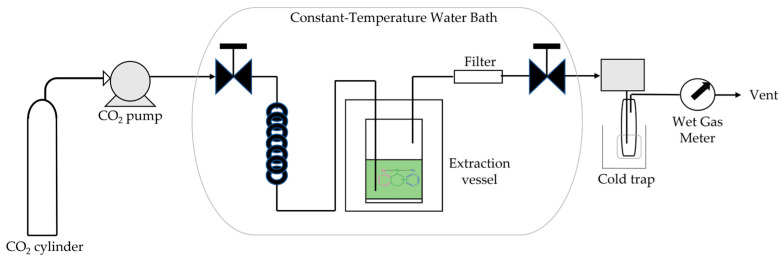
Schematic model of the supercritical fluid extraction apparatus.

Supercritical fluid extraction (SFE) uses fluids in conditions above their thermodynamic critical point of temperature and pressure. The density of supercritical fluids is similar to that of liquids; their viscosity is low, resulting in high diffusivity, and these properties enable supercritical fluids to penetrate more easily into solid compounds [8,9,119]. Although it requires specific and more expensive equipment, SFE is also considered a viable method, particularly when the extraction process needs higher selectivity (Figure 13).

Buelvas-Puello et al. found that SFE can be a suitable extraction method to obtain flavonoids from mango kernel, which showed consistent antioxidant activity. These extracts modified the oxidative stability of edible sunflower oil without adding other antioxidants. The total flavonoid content was equal to or greater than that obtained by Soxhlet extraction [120]. Végh et al. developed SFE to coextract sesquiterpene lactones and lipophilic flavonoids from the leaves of *Tanacetum parthenium* L. Twelve flavonoid components (including apigenin and luteolin) were detected in the extract, and eight additional methylated flavonoids were identified [121]. 

#### 5.3.4. Matrix Solid-Phase Dispersion Extraction

The matrix solid-phase dispersion extraction technique consists of three main steps. An additional solid-phase extraction clean-up step can be carried out by adding a cosorbent to the bottom of the extraction column or using different external columns [8,122,123]. The extract was evenly dispersed throughout the extraction column. The critical parameters were (i) the ratio of sample to solid material, (ii) the choice and composition of the eluent, and (iii) the type of dispersant material [122]. Mansur et al. developed a method based on matrix solid-phase dispersion extraction, compared to ultrasound-assisted extraction and homogenate-assisted extraction, to obtain flavonoids from common buckwheat sprouts and Tartary buckwheat sprouts. They showed that the main flavonoids of common buckwheat sprouts were extracted with significantly higher yields using the developed method than by the other mentioned techniques [124].

#### 5.3.5. Pulsed Electric Field Extraction

**Figure 14:**
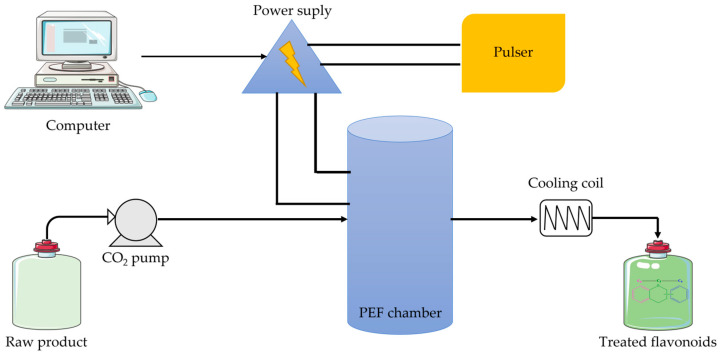
Schematic model of the pulsed electric field extraction apparatus.

The pulsed electric field extraction technique is a modern technique in which a very short voltage pulse with high electric field strength is applied to a biomaterial located between two electrodes, causing permeabilization and destruction of cell membranes by electroporation [8,9,125]. This method is recommended primarily in combination with other extraction techniques (Figure 14). 

Kim et al. determined the optimal extraction conditions of the flavonoid quercetin from dried onion skin using the pulsed electric field technique as a pretreatment for the subcritical water extraction method [126]. Manzoor et al. reported that a combination of pulsed electric field and ultrasound extraction techniques can be an alternative in food processing industries. The results showed an improvement in the total phenolic content, the total flavonoid content, and the antioxidant activity of almond extract [127]. 

#### 5.3.6. Enzyme-Assisted Extraction

Enzyme-assisted extraction is a pretreatment technique that uses specific enzymes to disrupt the cell wall of the source material to improve extraction yield [8,128]. It can be combined with various other techniques to enhance the overall recovery of bioactive compounds from different biomaterials. Granato et al. used a mixture of pectinases, cellulases, beta-1-3-glucanases, and pectin lyases to recover anthocyanins and polyphenols from blackcurrant press cake. The optimal extraction conditions were a solid:solvent ratio of 1:10 and 1:4 *w*/*v*, pH 5.5, while the temperature was chosen according to the type of enzyme—50 °C for cellulases and 40 °C for pectinases [129]. Amulya et al. optimized the extraction conditions by using the response surface methodology and central composite design for the recovery of anthocyanin pigments by enzyme-assisted extraction from eggplant peel. The best enzyme-assisted extraction parameters were a temperature of 37.32 °C, 5% enzyme concentration, and 1 h extraction time. This extraction technique was recognized as an effective way to extract bioactive compounds from eggplant peel [130]. 

Table 2 summarizes some of the most recent reports for the extraction of important flavonoids from plants using modern extraction techniques.

## 6. Outlook and Perspectives 

Flavonoids are important secondary metabolites produced by plants and microorganisms with several biological activities. Awareness of the biological properties of flavonoids has triggered increasing interest in flavonoids’ uses in medical, pharmaceutical, cosmetic, food, and/or nutraceutical industrial processes. Current trends in research and development activities on flavonoids relate to the identification, extraction, new functions, and applications of flavonoids for health benefits. Molecular docking, combined extraction methods, and inclusion of flavonoids in various delivery systems are also used to obtain larger amounts of flavonoids, higher solubility, and stability, allowing the development of new industrial manufacturing technologies. 

One of the main limitations blocking the broader use of flavonoids is their low bioavailability and solubility, poor absorption, and rapid metabolism. One of the future solutions to address these flavonoid limitations is the widespread use and development of nanotechnology.

Nanotechnology offers opportunities in all areas of scientific research, such as medical chemistry, medicine, and pharmaceutical science. The properties of nanoparticles, such as their small size and high surface, make them the best approach in the medical and pharmaceutical fields. They are able to improve the effectiveness of products extracted from plants, increase the yield of secondary plant metabolites relative to biomass, reduce adverse effects, and increase bioavailability. 

**Figure 15:**
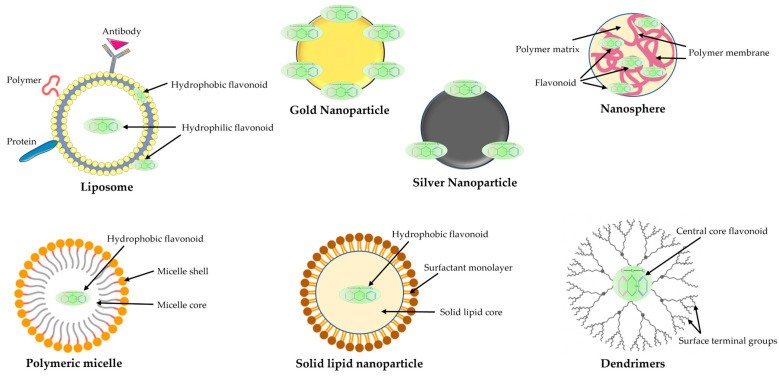
Inclusion possibilities of flavonoids in various delivery nanosystems.

Nano-vesicle systems (e.g., liposome and ethosome), micro- and nanoparticles, solid lipid nanoparticles, nanostructured lipid carriers, nanomicelles, and cyclodextrins, and dendrimers are some of the most common biocompatible and biodegradable nanoparticles [151,152,153]. The main flavonoid delivery systems are illustrated in Figure 15. 

For example, a recent review by Manocha et al. focused on nanonutraceuticals with enhanced bioavailability, solubility, stability, improved encapsulation, and sustained and targeted delivery with enhanced therapeutic activity. Nanotechnology has the potential to increase the stability and control of encapsulated flavonoids and non-supplements against natural changes, and nanoparticles offer promising potential as nutraceutical transporters [154].

Another study discusses the unique properties of nanomicelles for efficient delivery and improved bioavailability of various nutrients, such as flavonoids. Nanomicelles have several advantages due to their size and structural composition, increase the stability of drugs, protect them against elimination by the mononuclear phagocyte system, and lead to prolonged blood circulation [155].

A study by Sysak et al. revealed multiple synthesis possibilities for flavonoid–metal nanoparticle conjugates (e.g., silver nanoparticles and gold nanoparticles) and hybrids (metal oxide nanoparticles), also reviewing their characterization, biological properties, and medical applications [156].

The applications of nanotechnology for the targeted delivery of flavonoids to improve their bioavailability are beyond doubt. However, until now, such flavonoid delivery systems have been largely replicated in vitro and to a lesser extent in human models. In the near future, clinical trials could greatly contribute to improving the effectiveness and safety of using flavonoids as new treatment methods for human diseases, as well as to further the development of the medical and pharmaceutical fields.

The development of the production of flavonoids and their use for medical purposes will certainly be connected with overcoming the actual drawbacks and ensuring an optimal path from improved biosynthesis in plants (or engineered microorganisms) to extraction, clinical trial, and therapeutic use. At present, each of these aspects shows promising perspectives, but a consistent research effort is still needed to change the actual status of flavonoids from mostly dietary supplements to authorized drugs.

## Figures and Tables

**Table 1:** The basic skeleton of the various classes and biological activity of flavonoids.

Classification	Flavonoid	Biological Activity	References
**Flavanones** 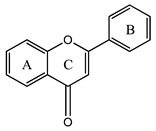	**Naringenin** (5,7-dihydroxy-2-(4-hydroxyphenyl)-chroman-4-one)	Antifibrinolytic, decreases pro-inflammatory cytokines in hepatocytes, antineoplastic activity (breast cancer, prostate cancer, lung cancer, and colon cancer), a promising treatment strategy against COVID-19, and therapeutic application in bone disorders and bone tissue engineered constructs	[11,12,13,14,15,16,17,18,19,20,62,63]
	**Hesperitin** ((2S)-5,7-dihydroxy-2-(3-hydroxy-4-methoxyphenyl)-2,3-dihydrochromen-4-one)	Anti-diabetic, antioxidant, anti-inflammatory, anticancer, antiviral, antibiofilm, antimicrobial, and cardiovascular protective activities	[21,22,23,24,25,64]
	**Eriodictyol** (2-(3,4-dihydroxyphenyl)-5,7-dihydroxy-2,3-dihydrochromen-4-one)	Cardiotonic, skin protection, antitumoral, antioxidant, anti-inflammatory, immunomodulatory, and hepatoprotective	[26,27,28]
	**Silymarin** (3,5,7-trihydroxy-2-[3-(4-hydroxy-3-methoxyphenyl)-2-(hydroxymethyl)-2,3-dihydro-1,4-benzodioxin-6-yl]-2,3-dihydrochromen-4-one)	Antioxidant, anti-inflammatory, and anticancer	[65]
**Flavones** 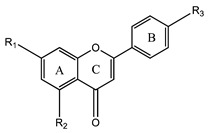	**Apigenin** (5,7-dihydroxy-2-(4-hydroxyphenyl)-chromen-4-one)	Neuroprotective	[32]
	**Baicalein** (5,6,7-trihydroxy-2-phenyl-4H-chromen-4-one)	Neuroprotective, antiviral activity, possible treatment agent against SARS-Cov2	[31,33,66]
	**Wogonin** (5,7-dihydroxy-8-methoxy-2-phenyl-4H-chromen-4-one)	Neuroprotective	[31,33]
	**Luteolin** (2-(3,4-dihydroxyphenyl)-5,7-dihydroxychromen-4-one)	Anti-inflammatory, antiallergy, and anticancer	[67]
	**Chrysin** (5,7-Dihydroxy-2-phenyl-4H-chromen-4-one)	Neuroprotective, anti-aging, skin protective, antioxidant, anti-inflammatory, anticancer, anti-diabetic, vasodilatory effect, and antihypertensive	[68,69,70,71]
	**Acacetin** (5,7-dihydroxy-2-(4-methoxyphenyl)-chromen-4-one)	Antiproliferative, neuroprotective, cardioprotective, anticancer, anti-inflammatory, anti-inflammatory diabetic, antimicrobial	[72]
	**Diosmetin** (5,7-dihydroxy-2-(3-hydroxy-4-methoxyphenyl)-chromen-4-one)	Antioxidant, anti-inflammatory, anti-apoptotic, and anticancer properties	[73]
**Isoflavones** 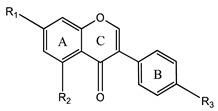	**Genistein** (5,7-dihydroxy-3-(4-hydroxyphenyl)-chromen-4-one)	Fungistatic, antibacterial, antiviral, anti-inflammatory, kidney protective, antioxidant, prevents angiogenesis, exerts estrogenic and/or antiestrogenic effects, and promising therapeutic application in bone disorders and bone tissue engineered constructs	[34,38,39,63,74,75,76,77]
	**Daidzein** (7-hydroxy-3-(4-hydroxyphenyl)-chromen-4-one)	Fungistatic, antibacterial, antiviral, antioxidant, prevents angiogenesis, exerts estrogenic and/or antiestrogenic effects, and promising therapeutic application in bone disorders and bone tissue engineered constructs	[34,38,39,63,77,78,79]
	**Glycitein** (7-hydroxy-3-(4-hydroxyphenyl)-6-methoxychromen-4-one)	Fungistatic, antibacterial, antiviral, antioxidant, prevents angiogenesis, and exerts estrogenic and/or antiestrogenic effects	[34,38,39,80,81]
	**Formononetin** (7-hydroxy-3-(4-methoxyphenyl)-chromen-4-one)	Fungistatic, antibacterial, antiviral, antioxidant, anti-inflammatory, neuroprotective, prevents angiogenesis, and exerts estrogenic and/or antiestrogenic effects	[34,38,39,82,83,84]
**Flavonols** 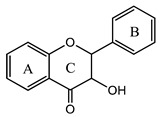	**Quercetin** (3,3,4,5,7-pentahydroxyflavone)	Downregulates malondialdehyde level and scavenges several free radicals (e.g., hydrogen peroxide, superoxide, and hydroxyl radicals), increases cell IFN-γ expression, decreases interleukine-4-positive cell expression, induces extrinsic and intrinsic pathways of apoptosis and autophagy, antimicrobial activity, inhibits biofilm formation, inhibits nucleic acid synthesis, antiviral activity, and possible treatment against SARS-Cov2	[41,42,43,44,45,66]
	**Rutin** (3,3′,4′,5,7-pentahydroxyflavone-3-rhamnoside)	Anti-inflammatory, anti-hypolipemiant, anti-atherosclerotic, antiallergic, anti-inflammatory, and antiviral	[46,47,48,49]
	**Morin** (2-(2,4-dihydroxyphenyl)-3,5,7-trihydroxychromen-4-one)	Antioxidant, anti-inflammatory, anticancer, anti-diabetic, anti-inflammatory, antihypertensive, and gastric protector effects	[85]
	**Kaempherol** (3,5,7-trihydroxy-2-(4-hydroxyphenyl)-chromen-4-one)	Antioxidant, antimicrobial, and anti-inflammatory	[86,87]
	**Myricetin** (3,5,7-trihydroxy-2-(3,4,5-trihydroxyphenyl)-chromen-4-one)	Antioxidant, anti-inflammatory, anti-diabetic, anti-epileptic, anti-Alzheimer, anti-apoptotic, antithrombotic, neuroprotective, potential therapeutic agent against COVID-19, and hepatoprotective effect	[88,89,90]
**Flavanols** 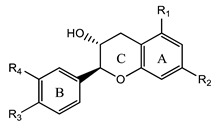	**Catechin** (2-(3,4-dihydroxyphenyl)-3,4-dihydro-2H-chromene-3,5,7-triol)	Anti-inflammatory, anticancer, antiviral, antimicrobial, and protective cardiovascular properties	[50,51,52,53]
	**Epigallocatechin** (2-(3,4,5-trihydroxyphenyl)-3,4-dihydro-2H-chromene-3,5,7-triol)	Anti-inflammatory, anticancer, antiviral, antifungal, antimicrobial, and protective cardiovascular properties	[50,51,52,53,66]
	**Afzelechin** (2-(4-hydroxyphenyl)-3,4-dihydro-2H-chromene-3,5,7-triol)	Anti-inflammatory, anticancer, antiviral, antimicrobial, and protective cardiovascular properties	[50,51,52,53]
**Anthocyanins** 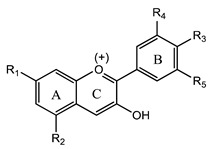	**Cyanidin** (2-(3,4-dihydroxyphenyl)-chromenylium-3,5,7-triol)	Immunomodulator and anti-inflammatory	[57,58,61]
	**Delphinidin** (2-(3,4,5-trihydroxyphenyl)-chromenylium-3,5,7-triol)	Anti-HER-2 effect, regulates the protein expression level of Bax and Bcl-2, inhibits the activation of NF-κB, induces G2/M phase arrest and apoptosis, pro-apoptotic, anti-proliferative effects, and anti-inflammatory	[59,60,61]
	**Pelargonidin** (2-(4-hydroxyphenyl)-chromenylium-3,5,7-triol)	Antioxidant and anti-inflammatory	[91,92,93]
	**Petunidin** (2-(3,4-dihydroxy-5-methoxyphenyl)-chromenylium-3,5,7-triol)	Antioxidant and anti-MIRI effects	[92,93]
	**Malvidin** (2-(4-hydroxy-3,5-dimethoxyphenyl)-chromenylium-3,5,7-triol)	Hypoglycemic, hypolipidemic, gastroprotective, hepatoprotective, antiadhesive, and antibiofilm effects	[94,95,96,97]

**Table 2:** Summary of recent reports on modern extraction techniques of flavonoids.

Extraction Technique	Target Flavonoids	References
Microwave-assisted extraction (MAE)	Flavonol, catechins	[113,131]
Ultrasound-assisted extraction (UAE)	Flavonols (isoquercitrin, quercetin)	[132,133,134]
	Anthocyanins	[135]
	Flavones (methoxyflavones)	[136]
Supercritical fluid extraction (SFE)	Flavanone (pinocembrin)	[137]
	Flavonol (galangin)	[138]
	Flavones	[121,139]
Pulsed electric field extraction (PEFE)	Flavanones	[140]
Enzyme-assisted extraction (EAE)	Anthocyanins	[129,130,141]
	Flavones	[142,143,144]
UAE + deep eutectic solvents	Flavanones, flavonols	[145,146]
UAE + butylene Glycol	Flavonols (catechins)	[147]
MAE + deep eutectic solvents	Flavones (trifolin, isoquercetin, kaempferol), flavonols (astragalin, quercetin, hyperoside)	[148]
UAE + EAE	Anthocyanins	[149]
MAE + ionic liquids	Flavones	[150]

## Data Availability

Not applicable.

## Abbreviations

The following abbreviations are used in this manuscript:

ABTS	2,2’-azino-bis(3-ethylbenzothiazoline-6-sulfonic acid)
ADT	Arogenate-dehydratase
C4CoAL	4-coumarate-coenzymeA-ligase
C4L	Cinnamate-4-hydroxylase
CoA	Coenzyme A
GAE	Gallic acid equivalent
IC_50_	Half maximal inhibitory concentration
IFN	Interferon
LDL	Low-density lipoprotein
PhAAL	Phenylalanine-ammonia lipase
PhAT	Prephenate-amino-transferase
QE	Quercetin equivalents
TE	Trolox equivalent antioxidant capacity
